# Spermatogonial stem cell technologies: applications from human medicine to wildlife conservation^†^

**DOI:** 10.1093/biolre/ioae109

**Published:** 2024-07-12

**Authors:** Katerina B Damyanova, Brett Nixon, Stephen D Johnston, Andrés Gambini, Patricio P Benitez, Tessa Lord

**Affiliations:** Discipline of Biological Sciences, The University of Newcastle, Callaghan, NSW 2308, Australia; Infertility and Reproduction Program, Hunter Medical Research Institute, New Lambton Heights, NSW 2305, Australia; Discipline of Biological Sciences, The University of Newcastle, Callaghan, NSW 2308, Australia; Infertility and Reproduction Program, Hunter Medical Research Institute, New Lambton Heights, NSW 2305, Australia; School of Environment, The University of Queensland, Gatton, QLD 4343, Australia; School of Veterinary Science, The University of Queensland, Gatton, QLD 4343, Australia; School of Veterinary Science, The University of Queensland, Gatton, QLD 4343, Australia; School of Agriculture and Food Science, The University of Queensland, Gatton, QLD 4343, Australia; School of Agriculture and Food Science, The University of Queensland, Gatton, QLD 4343, Australia; Discipline of Biological Sciences, The University of Newcastle, Callaghan, NSW 2308, Australia; Infertility and Reproduction Program, Hunter Medical Research Institute, New Lambton Heights, NSW 2305, Australia

**Keywords:** spermatogonial stem cells, male infertility, wildlife conservation, marsupials, stem cell technologies, spermatogonial transplantation, testis grafting, in vitro spermatogenesis

## Abstract

Spermatogonial stem cell (SSC) technologies that are currently under clinical development to reverse human infertility hold the potential to be adapted and applied for the conservation of endangered and vulnerable wildlife species. The biobanking of testis tissue containing SSCs from wildlife species, aligned with that occurring in pediatric human patients, could facilitate strategies to improve the genetic diversity and fitness of endangered populations. Approaches to utilize these SSCs could include spermatogonial transplantation or testis tissue grafting into a donor animal of the same or a closely related species, or *in vitro* spermatogenesis paired with assisted reproduction approaches. The primary roadblock to progress in this field is a lack of fundamental knowledge of SSC biology in non-model species. Herein, we review the current understanding of molecular mechanisms controlling SSC function in laboratory rodents and humans, and given our particular interest in the conservation of Australian marsupials, use a subset of these species as a case-study to demonstrate gaps-in-knowledge that are common to wildlife. Additionally, we review progress in the development and application of SSC technologies in fertility clinics and consider the translation potential of these techniques for species conservation pipelines.

## Teaser

Spermatogonial stem cell technologies developed for human infertility offer promise for wildlife conservation; however, bridging species-specific knowledge gaps is crucial for their application in rare and endangered species.

## Graphical Abstract

**Graphical Abstract:**
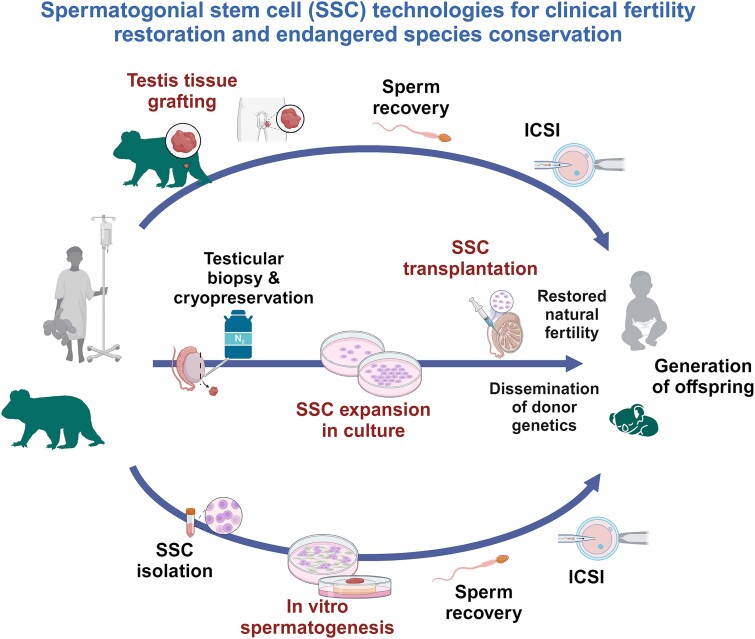

## Introduction

Spermatogenesis is a key function of the testis that ensures the continuity of sexually reproducing species through the daily production of millions of spermatozoa. Spermatogonial stem cells (SSCs) are a subpopulation of “type A” undifferentiated spermatogonia that form the basis of life-long spermatogenesis through their ability to balance self-renewal with the production of progenitor spermatogonia that are poised for differentiation. In addition to being indispensable for mammalian fertility, the unlimited self-renewal capacity of SSCs also holds exciting potential for the development of stem cell technologies aimed at human fertility preservation [1], conservation of vulnerable and endangered wildlife species, and enhancing animal production in agricultural settings [2]. In the realm of conservation, SSC biobanking technologies hold particular promise for species that produce sperm that is not yet amenable to cryopreservation, such as the Australian native marsupial, the koala [3].

This emphasis on the development of strategies to protect Australia's native marsupials is especially pertinent following the devastating 2019/2020 bushfire season. During this period, it is estimated that 1 billion animals were lost, including ~25,000 koalas. These events led to a 25% reduction in the koala population in New South Wales where the IUCN (International Union for Conservation of Nature) classification was changed from vulnerable to critically endangered [4]. Other contributing factors to the steep population decline are the high incidence of chlamydia infections, roadkill, and loss of habitat [5]. The potential loss of iconic Australian marsupial species such as the koala would have far-reaching consequences for biodiversity, along with negative socio-economic impacts. Thus, investment into development of a multitude of techniques aimed at protection and conservation of these species is warranted.

Despite the immense potential of SSC technologies, several roadblocks have delayed progress in their development, including a lack of knowledge on the basic biology of SSCs in non-rodent species, and *in vitro* approaches that inadequately replicate the microenvironment of the stem cell niche. Here, we discuss the potential use of SSCs in wildlife conservation and human medicine, focusing on the methodologies that would underly such technologies, including *in vitro* culture of undifferentiated spermatogonia, spermatogonial transplantation and xenotransplantation, and *in vitro* spermatogenesis. We also identify gaps in existing knowledge of SSC biology, particularly in marsupial species including the Australian koala (*Phascolarctidae*) and American opossum (*Didelphidae*) and explore how improved understanding of the mechanisms underpinning this self-renewing cell population could help overcome current limitations associated with SSC technologies.

## Defining characteristics of the SSC population across species

The dynamics of the undifferentiated spermatogonia population varies, even between closely related species, likely reflecting differences in intrinsic signaling pathways and extrinsic cues from the niche. This creates clear challenges in developing SSC technologies, in that certain requirements are likely to be species-specific. However, as we explore below, across (mammalian) species whose spermatogonia have been well characterized, there does tend to be a hierarchy of consistently observed spermatogonial sub-populations that express a handful of conserved genes and possess similar growth factor requirements and downstream signaling pathways. This provides us with a framework to understand the fundamental requirements for SSC function that can be built upon for species-specific application.

### Histological characteristics of SSCs and their differentiating counterparts

**Figure 1:**
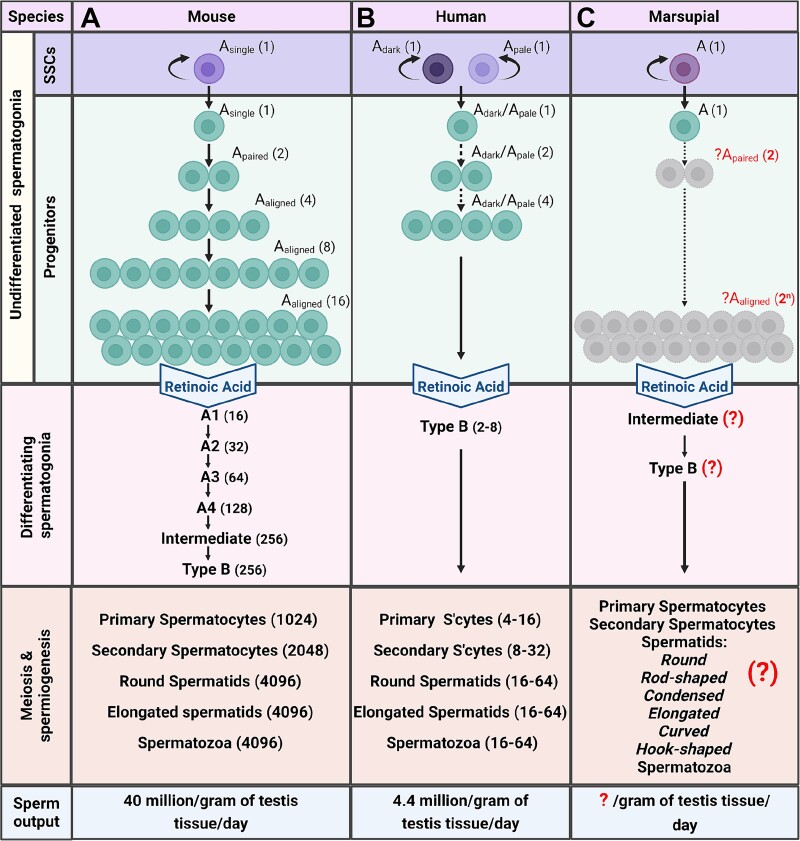
**Spermatogonia population dynamics in mouse, human and marsupials.** (A) In rodents, the revised A_single_ model illustrates germline stem cell dynamics, where a subset of A_single_ spermatogonia constitutes the SSC population. Transitioning into a progenitor state, these undifferentiated spermatogonia undergo incomplete cytokinesis, forming pairs (A_paired_) and chains (A_aligned_). Progenitors commit to differentiation in response to retinoic acid, transitioning into A1, then A2, A3, A4, Intermediate and Type B spermatogonia through successive mitotic divisions, before entering meiosis at the spermatocyte stage. Haploid spermatids resulting from meiosis undergo morphological changes during spermiogenesis to ultimately form mature spermatozoa. (B) In humans (A_pale_/A_dark_ model), undifferentiated spermatogonia may undergo one or two successive mitotic divisions as progenitors or transition directly into Type B differentiating spermatogonia upon the retinoic acid pulse without clonal expansion. (C) Much remains unknown about SSC dynamics in marsupials (designated with question marks). It is unclear if undifferentiated type A spermatogonia undergo transit-amplifying divisions before transitioning into Intermediate and Type B differentiating spermatogonia. Spermiogenesis in several Australian marsupials exhibits unique morphological changes in the haploid spermatids, differing from placental animals.

In the adult testis, spermatogonia are broadly classified as either undifferentiated or differentiating. Population dynamics and morphological characteristics of germ cells at different stages of differentiation have been well defined in laboratory rodents and some primates [6–8]; however, these characteristics remain elusive in most wildlife species, with a handful of exceptions including the feral pig [9, 10], and some reptile [11] and teleost [12–15] species. Although considerable interspecies variation exists, stem cells generally represent a rare subpopulation of spermatogonia in the testis (0.03% of total germ cells in the mouse [16]). In the mouse testis, SSCs have traditionally been considered to be “A_single_” (A_s_) spermatogonia [16, 17] that undergo mitosis to either produce two new A_s_ cells, or A_paired_ (A_pr_) spermatogonia that remain interconnected by cytoplasmic bridges. A_pr_ spermatogonia will then progress through further divisions in synchrony to produce chains of 4, 8, 16, and 32 A_aligned_ (A_al_) spermatogonia (Figure 1) [18, 19]. While some heterogeneity and fluidity is likely to exist within the sub-populations (see [20] for a comprehensive review), for the most part, clones of A_pr_ and A_al_ are thought to have substantially reduced self-renewal capacity and represent the progenitor cell population. In alignment with this, upon receiving the retinoic acid pulse in stage VII of the spermatogenic cycle, over 95% of A_pr_ and A_al_ spermatogonia will make the A-to-A1 (differentiating) transition, compared to less than 10% of A_s_ spermatogonia [16]. Following this, differentiating spermatogonia will transition further to A2, A3, A4, Intermediate, and Type B spermatogonia, before eventually forming spermatocytes.

Conversely, in the testis of nonhuman primates and humans, two distinct types of undifferentiated spermatogonia have been identified termed A_dark_ and A_pale_, based on their nuclear morphology and intensity of hematoxylin staining [21]. While functional evidence is sparse, both A_dark_, and A_pale_ are thought to possess stem cell activity, with A_dark_ spermatogonia being the reserve, non- or slow- cycling stem cells, while the A_pale_ cells actively proliferate to maintain the stem cell pool and fuel spermatogenesis [6, 22, 23]. Interestingly, while single-cell RNA sequencing (scRNAseq) analyses have revealed various spermatogonial states characterized by their transcriptional profiles [24–26], other investigations have found no significant differences in the transcriptomes of A_pale_ and A_dark_ spermatogonia [23, 27]. In contrast to rodents, the progenitor cells generated through A_dark_/A_pale_ division in humans and nonhuman primates undergo substantially fewer transit amplifying divisions (0, 1, or 2) [28, 29], resulting in a markedly reduced pool of differentiating spermatogonia. Subsequently, type B spermatogonia in the rhesus macaque undergo four transit amplifying divisions (B1-B4) prior to spermatocyte formation, compared to a single generation of type B spermatogonia in human [30–32].

While spermatogonial dynamics in laboratory rodents and some primate species have been relatively well defined, for most wildlife species, little to nothing is known about SSC behavior and the presence of different spermatogonial subsets. As our laboratory has a particular interest in marsupial conservation, we reviewed the literature on spermatogonial biology to provide a “case study” on this topic, and reveal the gaps-in-knowledge that will need to be filled before SSC-based technologies can come to fruition. In our search for information on marsupial spermatogonia specifically, as opposed to spermatozoa or other downstream germ cells, literature was limited to histological analyses in the Australian native koala (*Phascolarctos cinereus*) and southern hairy-nosed wombat (*Lasiorhinus latifrons*) [33], and primary cell culture [34] and single nucleus RNA sequencing (snRNA-seq) analysis on the grey short-tailed opossum (*Monodelphis domestica*) [35], which will be discussed in a subsequent section. Investigations into the koala and wombat seminiferous epithelium have revealed the presence of type A, Intermediate and type B spermatogonia [33], however, the complexities of the undifferentiated population are yet to be explored. For example, it is undetermined whether Type A spermatogonia in these marsupials remain connected by cytoplasmic bridges following division, and the magnitude of their clonal expansion (Figure 1). Another study examined the cellular associations and length of the cycle of seminiferous epithelium in four species of Australian marsupials: the tammar wallaby (*Macropus eugenii*), red-necked wallaby (*Macropus rufogriseus*), common brushtail possum (*Trichosurus vulpecula*) and long-nosed bandicoot (*Perameles nasuta*), finding similarities to eutherian mammals, but lacking characterization of pre-meiotic stages of germ cell development [36]. This underscores the gap in our understanding of spermatogonial dynamics in marsupials, a crucial aspect for their application in conservation approaches [37, 38].

### Molecular signatures of SSCs

**Figure 2:**
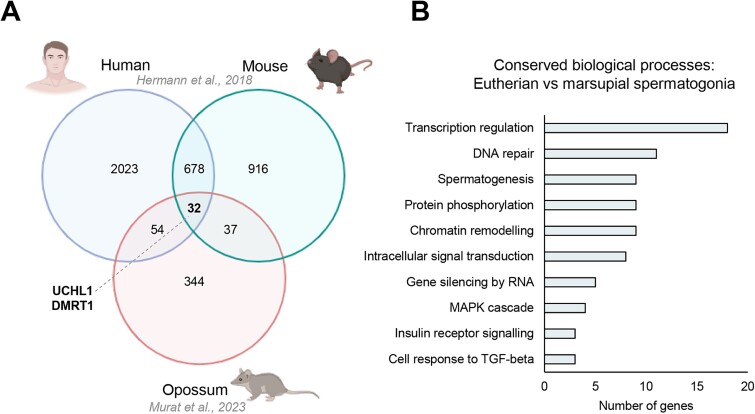
**Comparison of single cell RNAseq data from human, mouse and opossum SSCs.** (A) Venn diagram depicts the number of shared and unique differentially expressed genes in human and mouse undifferentiated spermatogonia (Hermann et al., 2018), and opossum spermatogonia (Murat et al., 2023). Thirty-two genes were common across all three datasets, representing putative conserved markers of SSCs between these species (also see Supplemental Dataset S1). (B) Conserved biological processes between eutherian and marsupial spermatogonia identified by gene ontology analysis (see Supplemental Dataset S1 for complete analysis).

Advancements in transcriptomic and proteomic technologies have facilitated the curation of molecular signatures in rodent and primate models that can, at least partially, delineate SSC populations from progenitor and differentiating spermatogonia. These molecular signatures include cell surface growth factor receptors, however, are predominantly comprised of transcription factors acting as master regulators of cell fate. Below, we offer a brief summary of well-established SSC markers, with comprehensive reviews available elsewhere [31, 39, 40]. Additionally, to shed light on surface markers, signaling pathways, and transcription factors that are likely to be conserved between eutherian and marsupial spermatogonia, we have mined the limited transcriptomic data available on marsupial spermatogonia. Specifically, we used the grey short-tailed opossum (*M. domestica*) snRNA-seq testis dataset recently produced by Murat, et al. [35] (E-MTAB-11072), obtained spermatogonia-marker genes, and compared them with undifferentiated spermatogonia markers identified via mouse and human testis scRNA-seq [41]; GSE109033 (adult mouse spermatogenic cells) and GSE109037 (adult human spermatogenic cells) (Figure 2). While informative, this comparison has limitations due to differences in annotation, sequencing depth, spermatogonial cell number and differentiation stage between the datasets. Thus, while we cannot place too much onus on the unique genes in each dataset, it is nonetheless interesting to assess those that are conserved to understand the fundamental requirements of SSC biology across species.

#### Surface markers identifying SSCs

In looking through the lens of developing SSC technologies for wildlife conservation and fertility treatments, the implications of identifying cell surface markers that are specific for the SSC population are two-fold. Firstly, the identification of unique surface receptors can allude to potential growth factors that are normally supplied by supporting niche cells that would need to be supplemented to sustain self-renewal capacity *in vitro* (discussed further below). Secondly, unique surface markers provide a target for antibody-based isolation strategies (fluorescence activated cell sorting (FACS) or magnetic activated cell sorting (MACS)) for these cells. Given the rarity of SSCs, particularly in the adult testis, this is an important component of any future biobanking pipelines.

A recent comprehensive review by Song et al. [42] has provided a list of common molecular markers of undifferentiated spermatogonia in vertebrates and their reported cellular localization. Among these, the most abundant cell surface proteins across different mammalian species were the glial cell line-derived neurotrophic factor (GNDF) receptor alpha 1 (GFRA1), integrin subunit alpha 6 (ITGA6), integrin subunit beta 1 (ITGB1), and thymocyte differentiation antigen 1 (THY1) or CD90. Importantly, the authors confirmed the evolutionary conservation of these genes across bovine, feline, porcine, canine, rodent, and primate species, while others have also reported GFRA1 as a stem cell-specific marker in the collared peccary [9, 10], scorpion mud turtle [11] and some fish species [12–14]. Other cell surface markers that have been used for SSC enrichment in the mouse are Cadherin 1 (CDH1) [43], TSPAN8 [44], CD9 [45], CD2 [46], melanoma cell adhesion molecule (MCAM) [47], epithelial cellular adhesion molecule (EpCAM) [48], and ephrin type-A receptor 2 (EPHA2), [49] although the conserved nature of these is less well defined. These markers have been employed to enrich SSC populations with varying degrees of success, as assessed by a quantitative spermatogonial transplantation assay (Table 1). The most effective SSC enrichment reported was 561-fold, achieved through multiparameter flow cytometric selection where CD9^+^EPCAM^low^MCAM^+^KIT^−^ phenotype exhibited the highest frequency of SSCs (one in every six cells) [47].

**Table 1:** Surface markers used for SSC enrichment in mouse testis suspensions via FACS or MACS

Surface marker	Enrichment compared to unselected control	Reference
Gfrαl	0.13 to 2.5-fold	[252]
α6 integrin	6-fold	[253]
β1 integrin	3-fold	[253]
Thyl	30-fold	[75]
CD9	7-fold	[40]
	32-fold	[43]
Cdh1	3.47 colonies/10^4^ cells compared to 0 in CDH1^neg ^population	[38]
EpCAM	3-fold	[43]
MCAM	0.62-fold of MCAM-overexpressing cells compared to wild type	[42]
Tspan8	2-fold	[39]
CD2	292-fold	[41]
EphA2	258-fold via FACS - EphA2^high^ vs control (4-fold via MACS)	[44]

**Figure 3:**
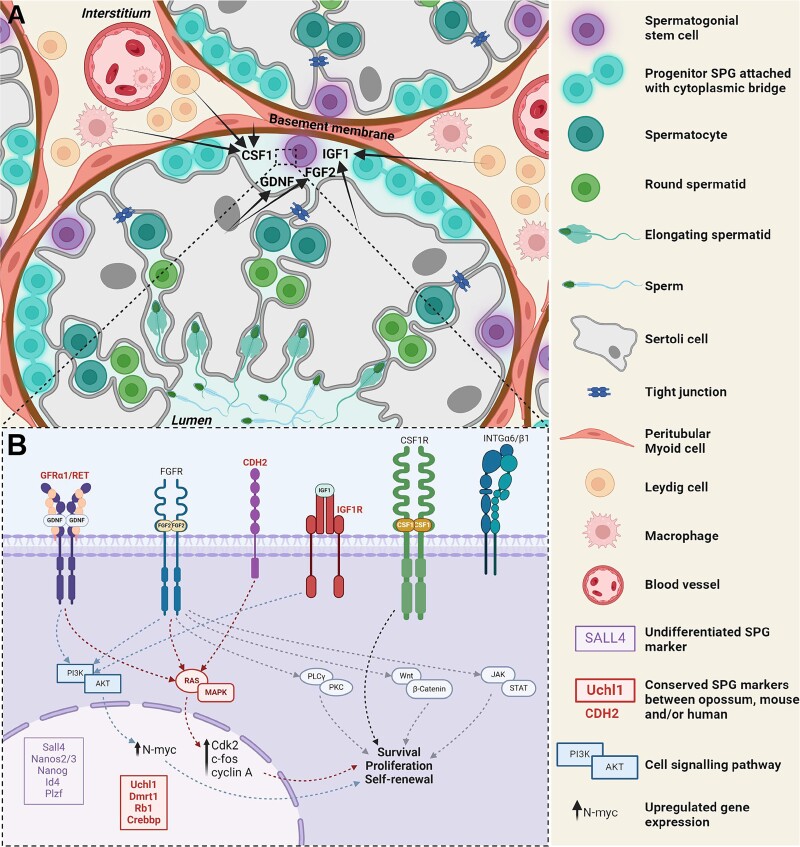
**Growth factor signaling in the SSC niche.** (A) The SSC niche is an “open” niche consisting of spermatogonia and surrounding somatic support cells, including Sertoli, Leydig, and PTM cells. The somatic cells release growth factors to promote maintenance and self-renewal of SSCs (arrows) including Sertoli cell-derived GDNF and FGF2; CSF1 from Leydig cells, myoid cells and interstitial macrophages; and IGF1 from Leydig cells. (B) GDNF binds to the GFRA1/RET receptor complex to activate multiple signaling cascades, including the PI3K/Akt pathway via SRC kinase recruitment, leading to N-myc expression and SSC proliferation. Concurrently, it triggers rapid and transient Ras/MAPK pathway activation, upregulating cell cycle activator genes (*Cdk2, c-fos, cyclin A*). FGF2 binding to FGFRs induces receptor tyrosine kinase dimerization and phosphorylation, activating various pathways (PI3K/Akt, Ras/MAPK, PLCγ/PKC, JAK/STAT, Wnt/β-catenin), modulating genes involved in undifferentiated spermatogonia survival, growth, and proliferation. The IGF1-IGF1R interaction also activates the PI3K/Akt pathway, enhancing SSC survival and stem cell capacity. SCF1R activation by CSF1 also promotes SSC self-renewal and proliferation. CDH2 and INTGa6/b1 are important for cell–cell and cell-extracellular matrix interactions that are crucial for SSC function. Cell surface markers, signaling pathways and genes that we found to be conserved between the opossum, mouse and/or human are indicated in red. Figure created using BioRender.

Clearly, the level of conservation of molecular markers can diminish as the evolutionary distance between species increases. Therefore, to use SSC-specific markers for cell isolation, the extent of conservation of these genes needs to be established in less well-characterized species. For instance, considerable differences in SSC marker expression are noted even with closely related species such as the mouse and rat [45, 48, 50]. To begin to ascertain which cell surface markers are likely to be conserved in marsupial spermatogonia, we mined the scRNA-seq list of spermatogonia identifiers for the grey short-tailed opossum recently produced by Murat et al. [35] (Figure 2, Supplemental Dataset S1). While this does not provide an exhaustive list of expressed cell surface proteins, it identified the conserved expression of GFRA1 and insulin-like growth factor 1 (IGF1). Interestingly, CDH2 or N-Cadherin, a paralog of CDH1 used as a surface marker for SSC enrichment in the mouse, was also identified. While CDH2 is known for its roles in gonad differentiation, testis morphogenesis and function [51] as well as survival and differentiation across various stem cell lineages [52], it has not been used for SSC enrichment in model species. On the other hand, a CDH2-knockout mouse study revealed an important role for the molecule in germ cell survival and maintenance during the early stages of spermatogenesis [51]. Altogether, this, along with its positive regulation of the MAPK cascade that is involved in survival, growth and proliferation of undifferentiated spermatogonia (discussed below, Figure 3), suggest that CDH2 might serve as a species-specific marker of SSCs in the opossum and potentially other marsupials.

#### Transcription factor expression delineating spermatogonial sub-populations

In overlaying spermatogonia markers from human, mouse and opossum scRNA-seq datasets, transcription factors were the most highly conserved category in terms of gene ontology analysis across the eutherian and marsupial spermatogonia (11% of genes), followed by those involved in DNA repair, chromatin remodeling, and “spermatogenesis” (Figure 2B). This is perhaps unsurprising, given that extensive studies in rodent models have highlighted the importance of transcription factor networks in the maintenance of the undifferentiated spermatogonia population (reviewed in [39]). Conserved transcription factors identified in our analysis included Ubiquitin C-Terminal Hydrolase L1 (UCHL1), doublesex- and mab-3-related transcription factor 1 (DMRT1), retinoblastoma protein 1 (RB1), and CREB binding protein (CREBBP) (Table 2). These factors have been previously defined to be pivotal in regulating SSC function in the mouse and/or human testis. Indeed, knockout of *Uchl1, Dmrt1,* or *Rb1* in mouse germ cells has been shown to result in progressive germ cell loss leading to azoospermia [53–55], while a large-scale siRNA screen on SSCs identified *Crebbp* as a candidate important for maintenance and self-renewal [56]. Confirming the conservation of these transcription factors between marsupial spermatogonia and the human/mouse is crucial for facilitating their downstream application in conserving rare and endangered species.

**Table 2:** Conserved transcription factors between opossum, human and/or mouse that regulate SSC function

Gene	Species expressed	Putative function	Reference
UCHL1	Humans, rodents, primates, felines, canines, livestock, opossums	Maintenance of SSC homeostasis, metabolism, and differentiation competence	[30, 36, 37, 48, 254]
DMRT1	Humans, rodents, primates, birds, reptiles, amphibians, fish, opossums	Maintenance of SSC homeostasis by regulating PLZF expression. Necessary in progenitor spermatogonia for replenishing the SSC pool after cytotoxic germ cell depletion.	[30, 36, 49, 255–259]
RB1	Humans, rodents, primates, birds, opossums	Influences fate determination of undifferentiated spermatogonia and self-renewal of SSCs through interaction with ID4.	[30, 36, 50, 260–262]
CREBBP	Humans, rodents, primates, opossums	Influences the expression of testis-specific transcription factors crucial for the in vitro maintenance of SSCs. Involved in chromatin remodeling.	[30, 36, 51, 262, 263]

#### Metabolism of SSCs

Although transcription factors undoubtably play a key role in regulating SSC maintenance, it has become apparent that metabolism is also an important dictator of stem cell fate decisions. Certainly, in other stem cell types, it has been shown that glycolytic metabolism is required for maintenance of a pluripotent state, while a metabolic shift to oxidative phosphorylation (OXPHOS) facilitates the differentiating transition [57, 58]. In alignment with this, a comprehensive analysis of recently published scRNA-seq data conducted by our own research group [59] revealed that expression profiles of genes related to glycolytic metabolism are higher in SSCs when compared to progenitors and differentiating spermatogonia from both mouse and human testes. Conversely, a shift towards differentiation was associated with higher expression of genes involved in mitochondrial biogenesis and OXPHOS. Although few functional assessments had been conducted at the time to ascertain whether this metabolic shift was an active contributor defining spermatogonial identity, multiple publications had demonstrated that driving glycolysis through overexpression of *Myc/Mycn* [60], or by reducing oxygen concentration to 10% [61] was consistently linked with increased self-renewal capacity. Encouragingly, since the publication of our 2020 review [59], a number of studies have produced additional data in support of this paradigm. For instance, a study conducted by Chen et al. [62] demonstrated that retinoic acid-induced differentiation of *in vitro* cultured mouse spermatogonia led to a reduction in glycolysis and an increase in OXPHOS. Additionally, data produced by Voigt et al. suggested that prospermatogonia in the embryonic testes preferentially utilize OXPHOS pathways, while a shift towards glycolysis occurred upon SSC formation, particularly post-puberty [63–65].

In considering whether a preference for glycolytic metabolism is conserved in marsupial SSCs, we were not able to infer this from existing data. Nevertheless, in the development of biobanking pipelines, the determination of metabolic requirements will be an important component to sustaining these cells *in vitro*. One crucial factor that may affect SSC metabolism is the basal metabolic rate (BMR) of different species, or the amount of energy expended by the organism while performing basic life-sustaining functions. Deviations in basal metabolism could have multiple impacts at the cellular level including altered energy demand, mitochondrial activity, oxidative stress (ROS production), cellular adaptation to environmental changes, and nutrient utilization [66, 67]. BMR, in turn, is affected by body size, food habits, climate, habitat, type of reproduction, and many other factors [68]. For instance, koalas feeding on nutritionally poor *Eucalyptus* foliage exhibit considerably lower metabolic rates than humans, who consume a diverse, energy-dense diet, or omnivorous pigs, known for their efficient feed conversion and rapid growth rates [69–71]. Therefore, significant differences in metabolic rate and energy demand could be expected between animals from different ecological niches. Given the important role of basal metabolism in shaping cellular metabolic adaptations [66, 72], it is reasonable to assume that there would be substantial interspecies variations in SSC metabolic activity, and therefore, niche requirements.

Further to this, the metabolic requirement of spermatogonia from different species could also vary depending on the size and dynamics of the undifferentiated population. As mentioned previously, the size of SSC and progenitor pools, and the number of transit-amplifying divisions in mice differ significantly to humans and non-human primates (Figure 1). This divergence results in variable spermatogenic efficiency, quantified by the daily sperm output per gram of testicular tissue, which markedly differs between mice (40 million sperm per gram of testicular tissue per day) and humans (4.4 million) [16, 31]. This is largely attributed to variations in the proliferative activity of spermatogonia between primates and non-primate mammals, with lower proliferative activity reducing metabolic strain (e.g., the amount of ATP required during steady state) [73, 74].

### Niche signals driving SSC fate

The testis microenvironment, or “niche”, within which spermatogonia reside is a key regulator of spermatogonial fate commitment [75]. Factors such as cytokines, adhesion molecules, and oxygen availability in the niche dictate intracellular signaling cascades, transcription factor expression, and metabolism that direct spermatogonial fate decisions. Thus, understanding niche signals is a key component in facilitating SSC maintenance *in vitro*, such as within biobanking pipelines for wildlife conservation, or in a clinical setting to reverse infertility.

#### Growth factor signaling

The processes of self-renewal and differentiation of stem cells needs to be precisely regulated through both intrinsic and extrinsic factors to prevent depletion of stem cells or reduced sperm production. Growth factor signaling orchestrated by somatic support cells in the niche (Sertoli, Leydig, peritubular myoid (PTM) cells, and macrophages), is instrumental in governing this balance (Figure 3). The most well characterized ligand-receptor interactions in undifferentiated spermatogonia are the Sertoli cell-secreted GDNF to GFRA1/RET [76–79], and fibroblast growth factors (FGF) 2 and 8 to FGFRs [80, 81]. Other examples include Sertoli cell-expressed chemokine (C-X-C motif) ligand 12 (CXCL12) to the G-protein coupled receptor CXCR4 [82, 83], colony stimulating factor 1 (CSF1) produced by peritubular macrophages, Leydig and PTM cells to CSF1R [84, 85], and the Leydig cell-secreted IGF1 to IGF1R [86].

GDNF binding to the GFRA1/RET receptor complex is responsible for the activation of multiple signaling cascades including the PI3K/Akt pathway and Ras/MAPK pathway, resulting in the recruitment of self-renewal driving transcription factors and the upregulation of cell cycle activator genes, promoting the proliferation and self-renewal of SSCs [87]. Similarly, FGF2 binding to FGFRs leads to dimerization and phosphorylation of the receptor tyrosine kinases resulting in recruitment of secondary messenger molecules and activation of numerous pathways, including PI3K/Akt, Ras/MAPK, PLCγ/Ca^2+^/PKC, JAK/STAT, Wnt/β-catenin and transforming growth factor beta (TGF-β) (reviewed in [88, 89]). Again, the downstream gene modulation regulates cell survival, growth, and proliferation of undifferentiated spermatogonia [81, 90].

In considering growth factor signaling pathways that are likely to be conserved in marsupial species, our gene ontology analysis of scRNA-seq data (Figure 2B) identified significant enrichment of MAPK signaling, insulin receptor (INSR) signaling, and cell response to TGF-β. The enrichment of MAPK and TGF-β signaling suggests that the main activators of these cascades (GDNF-GFRA1/RET and FGF2-FGFR1, respectively) likely play an instrumental role in the maintenance of the undifferentiated spermatogonia pool in the opossum testis. Furthermore, INSR and IGF1R signaling have been shown to contribute to the maintenance of mouse SSCs [81], including via activation of the PI3K/Akt pathway [86], thus suggesting that this pathway is similarly conserved to orchestrate SSC self-renewal in the opossum testis, and perhaps marsupial species more broadly. Identification of conserved signaling pathways between eutherian and marsupial SSCs represents a significant advancement in our comprehension of their fundamental biology.

#### Oxygen availability in the niche

In addition to the production of growth factors, stem cell niche microenvironments are also renowned for dictating cell fate through oxygen availability, usually regulated by the vicinity of different cell populations to the vasculature. This is an important consideration in the development of SSC technologies, particularly for the maintenance of these cells *in vitro* due to direct influences on metabolism and regenerative capacity. Two recent studies have used oxygen-sensing probes to demonstrate that, at least in the mouse testis, SSCs reside in conditions of <3% oxygen, while transition into a differentiating state is associated with increased oxygen availability [91, 92]. Our own studies have shown that modulation of hypoxia-inducible factor (HIF) pathways directly influences SSC function, with targeted HIF activation in undifferentiated spermatogonia *in vitro* instigating a significant increase in formation of colonies of spermatogenesis after transplantation into an infertile testis [92]. Mechanistically, this is likely to be primarily linked with the known capacity for HIFs to modulate cellular metabolism [92]. Importantly, the absence of information on oxygen availability in the testicular microenvironment of marsupial species hampers the characterization of their metabolic requirements and functional regulation, which are crucial aspects for the biobanking of spermatogonia.

## S‌SC technologies

**Figure 4:**
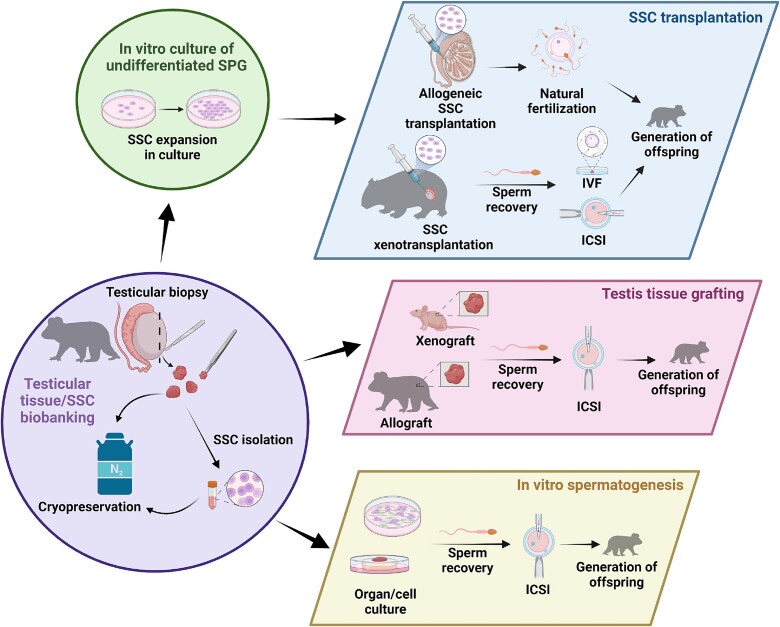
**Overview of conservation pipelines using SSC biobanking.** The proposed conservation pipelines for SSC biobanking begin with the collection and cryopreservation of testicular tissue or isolated SSCs from recently deceased endangered or vulnerable animals. Following expansion in culture, cryopreserved-thawed or fresh SSCs could be transplanted into the testis of another individual of the same species (allogeneic) to propagate donor genetics through natural mating. Alternatively, donor SSCs could be transplanted into a recipient from a closely related species (xenotransplantation) to facilitate in vivo spermatogenesis and sperm recovery for assisted reproduction approaches. Stored testis tissue could also be grafted onto an individual of the same species (allograft) or different species (xenograft), functioning as a bioincubator to facilitate *ex vivo* spermatogenesis and sperm recovery. Additionally, isolated SSCs or thawed testicular tissue could be cultivated in an environment conducive to stem cell differentiation and sperm production through in vitro spermatogenesis. The retrieved sperm could then be utilized via ICSI. Figure created using BioRender.

Enhancing our understanding of the molecular characteristics and functional regulation of SSCs across species holds substantial translational potential by laying the foundation for establishment of biobanking systems for wildlife conservation (Figure 4). Currently, spermatogonia preservation pipelines are in development with a primary focus on reversing human infertility in the clinic. However, successful development of SSC-based techniques in a clinical setting would also be directly applicable and adaptable for animal production and wildlife biobanking practices. Below, we explore current progress in spermatogonial biobanking (cryopreservation), *in vitro* expansion and differentiation, spermatogonial transplantation, and testis tissue grafting, and how these techniques might be applied in for mammalian conservation. Notably, while technologies involving induced pluripotent stem cells (iPSCs) hold significant potential in the space of human infertility treatment and wildlife conservation [93–98], they fall outside the scope of this review and will not be discussed further here.

### Development of clinical SSC-based technologies

The impetus to develop technologies that utilize testicular stem cells for fertility preservation has primarily been driven by a need in the context of pediatric oncology. With a marked increase in both the incidence and survival rates of childhood cancers in recent decades [99], there is a growing cohort of individuals that are faced with permanent loss of fertility as a result of exposure to cytotoxic therapies in childhood [100]. As sperm banking is not an option for prepubertal males, cryopreservation of SSCs captured in testicular tissue is the only possible alternative for these patients [101]. Proposed pipelines to use these SSCs include autologous transplantation post-recovery (likely following a period of *in vitro* SSC expansion), autologous grafting of testis tissue, or *in vitro* spermatogenesis to produce mature spermatozoa for use in assisted reproductive technologies (ARTs) (reviewed in [1]). Of these approaches, only spermatogonial transplantation holds the promise of restoring natural fertility, while the endpoints of tissue grafting and *in vitro* spermatogenesis would be to recover mature sperm that could be subsequently used for intracytoplasmic sperm injection (ICSI). Albeit, the transplantation of either reproductive tissues or cells back into the patient carries the risk of reintroducing malignant cells, particularly in the case of hematologic cancers, which are among the most prevalent pediatric malignancies [102], where cancer cells can infiltrate the testis via migration through the bloodstream [103]. Therefore, detection and depletion of cancer cells is an important consideration to avoid malignant relapse [104–106].

Although successful fertility restoration has not yet been achieved in humans, studies in non-human primates have demonstrated the feasibility of these procedures. For instance, allogeneic SSC transplantation in Rhesus macaques (*Macaca mulatta*), rendered infertile via alkylating chemotherapy, resulted in restoration of spermatogenesis and formation of embryos through ICSI [107]. In a separate study, mature spermatozoa were retrieved from cryopreserved prepubertal testicular tissue autologously grafted under the back or scrotal skin of pubertal Rhesus macaques, and were used to establish a pregnancy and generate healthy offspring (graft-derived baby “Grady”) [108]. Research involving human subjects has been limited, with only one study describing autologous transplantation of cryopreserved testicular cell suspensions into 11 Hodgkin lymphoma patients after cancer treatment, although no follow-up data have been published [109]. However, approval for clinical trials using these techniques has now been granted in the United States and United Kingdom, thus, progress is anticipated in this field in the coming years.

### Adapting clinical pipelines for SSC biobanking for wildlife conservation

As mentioned previously, SSC technologies that are currently in development in the clinic hold considerable potential to be adapted to contribute to conservation efforts for vulnerable and endangered species, through the preservation and reintroduction of genetic diversity. Such approaches would have particular utility for species whose sperm have not yet proven amenable to cryopreservation, such as the koala [3], kangaroos and wallabies [110], and a number of felid species [111, 112]. Additionally, cryopreservation of testicular tissue might be the only viable option for animals that have died unexpectedly (e.g., through disease(s) or road accidents) or prepubertal individuals, neither of which can produce spermatozoa through ejaculation. Crucial steps in the pipeline to use bio-banked wildlife testicular tissue may include the isolation and expansion of enriched populations of SSCs from the testis, spermatogonial transplantation, testicular tissue grafting, and/or *in vitro* spermatogenesis (Figure 4). Although the application of such SSC technologies might appear elaborate and ambitious, it is worth noting that similarly complex pipelines using bio-banked tissue and reproductive technologies have been successfully employed to rescue endangered wildlife species from the brink of extinction; such as the use of somatic cell nuclear transfer to rescue the black-footed ferret [113, 114].

### Limitations of current strategies for marsupial sperm biobanking

As mentioned above, suitable cryopreservation approaches are not currently available for the spermatozoa of several Australian marsupial species (e.g., koala, kangaroo, wallaby), creating unique challenges in captive breeding and biobanking. In the case of the koala, cryopreserved sperm experience head swelling upon thawing, loss of plasma membrane integrity, and loss of viability [3], creating a roadblock for their use in downstream assisted reproduction approaches. Although the underlying cause of this issue remains undefined, it has been postulated to be associated with an absence of cysteine residues in koala protamines [3] that normally facilitate chromatin stabilization through disulfide bond formation [115]. Since histone-to-protamine transition occurs at the round spermatid stage during spermatogenesis [116], the absence of protamines in the earlier germ cell stages suggests that koala SSCs may be more conducive to cryopreservation than their mature sperm. Beyond this, the challenges in marsupial sperm cryopreservation may also be related to the unusual morphological changes that occur during spermiogenesis and post-testicular maturation in some marsupials, contrasting with placental animals [33, 117, 118]. In addition, a notable advantage of biobanking of SSCs over spermatozoa is that these stem cells retain their recombination potential during meiosis. In contrast, the genetic variation of mature spermatozoa is confined to the number of cells present in the cryopreserved sample, representing a finite genetic source that is additionally impacted by reduced functional versatility (e.g., fertilizing potential, tolerance to cold-shock, etc.) as a result of the freeze–thaw process [119–121].

## Cryopreservation of testicular tissue and SSC populations

Cryopreservation methodologies are a crucial consideration in the design of biobanking pipelines for clinical and conservation applications. Freezing biological samples at extremely low temperatures effectively halts biochemical reactions, metabolic processes, and degradation, allowing for long-term storage and preservation of their integrity and functionality [122]. In conservation efforts, cryopreservation facilitates the utilization of biological materials collected from spatially or temporally distant populations [123].

Importantly, it has been demonstrated that mouse SSCs cryopreserved long-term in liquid nitrogen maintain their capacity to regenerate spermatogenesis after transplantation into an infertile testis and produce fertilization-competent spermatozoa and normal progeny [124, 125]. Conveniently, cryopreservation of SSCs is relatively straightforward and involves the use of freezing media containing 5–10% dimethyl sulfoxide (DMSO), fetal bovine serum (FBS), and/or bovine serum albumin (BSA) in cell culture media (e.g., MEM, DMEM) [124, 126, 127]. Furthermore, the addition of non-permeable agent (sucrose or trehalose) to the cryomedia along with the use of slow-rate freezing has improved post-thaw outcomes in both cryopreserved testicular tissue and isolated SSCs [128–131].

Protocols for testicular tissue and SSC cryopreservation have been successfully implemented not only in rodents and primates but also in humans [132, 133]; livestock species such as bovine, porcine, ovine, and goat [130, 131, 134–136]; as well as equine [137], feline [138], and teleost species [139–141]. In contrast, limited research exists on wildlife species, with a handful of studies attempting testicular tissue cryopreservation in species such as the jungle cat, lion, leopard, Rusa deer, Fea’s muntjac, Sumatran serow [142], Eld’s deer [143], barking deer, sambar deer, hog deer [144], Indian mouse deer [145], and gray wolf [146]. Notably, several of these studies lack rigorous assessment of post-thaw viability for the cryopreserved SSCs or testicular tissue. Functional evidence, including colonization capacity of recovered SSCs, production of mature fertilization-competent spermatozoa and normal offspring is critical for harnessing bio-banked material in both clinical and conservation settings. This is particularly pertinent given recent findings indicating compromised differentiation capacity of rat spermatogonia resulting from prolonged cryopreservation [127].

While considerable interspecies variation in tissue cryosensitivity has been observed, it has been argued that similarities between taxa enable a protocol developed for one species to offer valuable insights for another [147]. This underscores the necessity to generate new knowledge on the fundamental biology of under-researched species, such as most endangered wildlife, and emphasizes the importance of conducting comparative studies across diverse species [148].

## *In vitro* expansion of undifferentiated spermatogonia

Given that SSCs are an exceptionally rare population of cells [16] and that the success of SSC technologies relies on the availability of sufficient numbers of functional stem cells, a period of *in vitro* expansion is likely to be indispensable. This procedure, however, is not without challenges. For instance, successful SSC culture requires the isolation of relatively pure population of stem cells, given that contaminating somatic cells, such as fibroblasts, can rapidly outcompete and overwhelm the slow-cycling SSCs in a culture dish. Additionally, even established approaches to primary culture of undifferentiated spermatogonia in the mouse continue to suffer from significant losses in SSC function and content over time [61, 149], underscoring the need for further optimization of culture conditions.

### S‌SC harvesting and purification

Due to the scarcity of SSCs in the testes of adult animals, neonatal and prepubertal testes are the preferred source for harvesting this cell population for experimental purposes, as undifferentiated spermatogonia are the predominant cell type during these early developmental stages [150]. Alternatively, surgical cryptorchidism has also been employed for *in vivo* SSC enrichment in adult animals, with heat stress causing ablation of differentiating germ cell populations but leaving behind an unaffected undifferentiated spermatogonia pool (~20-fold enrichment compared to wild-type) [151]. However, when harvesting SSCs for biobanking pathways it will not be feasible to experimentally manipulate or select animals of a particular age, although this method could be valuable for identifying biomarkers in more common representative taxa. Thus, there is a critical need to develop effective SSC isolation techniques that can separate this rare population of cells from the abundance of other testicular cells. One such strategy is to identify SSC-specific surface markers that can be exploited via FACS or MACS approaches. Among the undifferentiated spermatogonia-specific markers currently used for “SSC selection” are UCHL1, CDH1, Promyelocytic leukemia zinc finger protein (PLZF), octamer-binding transcription factor 4 (OCT4), NANOG, ITGA6, and GFRA1 (Table 1).

In attempts to develop alternative strategies for SSC enrichment, the light scatter properties of SSCs from a handful of species (e.g., teleost, porcine) have also been characterized to facilitate their isolation by flow cytometry [152–154]. This approach obfuscates the need for SSC-specific surface proteins and highly selective antibodies, thus making it attractive for less studied species such as endangered wildlife. Similarly, enrichment approaches have also been developed that employ Staput velocity sedimentation [155] and differential plating, which incorporates different extracellular matrix components for SSC selection [80, 156, 157]. When used alone, velocity sedimentation and differential plating do not isolate highly pure populations of spermatogonia, however, in combination, these strategies significantly increase the number of putative SSCs.

### S‌SC expansion in culture

The robust expansion and survival of mouse SSCs in culture posed a significant challenge until the discovery that the growth factor GDNF could drive self-renewal of SSCs [158], as evidenced by a significant increase in ability to colonize recipient testes following transplantation when compared to non-supplemented control cultures [159]. This breakthrough led to the broader application of GDNF as a supplement for SSCs alone and in combination with other growth factors (e.g., FGF2) in both rodents and livestock species [160–163]. Despite this, the capacity to robustly maintain mouse SSCs long-term *in vitro* remains an ongoing challenge. Indeed, Helsel et al. [61] showed reduction in both stem cell number and regenerative capacity over 6 months of *in vitro* expansion. Although a reduction in oxygen concentration from 21% to 10% significantly improved regenerative capacity [61, 164], SSC function continued to decline after 4 months in culture. This underscores the need for ongoing refinement of SSC culture conditions, even in laboratory rodent models, particularly considering the limited starting material (scarcity of SSCs in the testes) requiring months of culture expansion to attain sufficient numbers of SSCs for fertility preservation and biobanking pipelines.

Despite some similarities, the direct transfer of culture conditions optimized in rodents to wildlife and human SSCs has not been possible without modifications. Differences in the metabolic requirements of SSCs during their prolonged prepubertal development phase in larger animals and humans, along with the influences of basal metabolism, diet, climate, and habitat could explain the necessity of altered culture systems to support these non-rodent stem cells [165–167]. A recent systematic review [168] evaluating successful *in vitro* SSC cultures ranging from 5–15 days to >1 month in livestock species (such as bovine, sheep, and goat), identified the persistence of several species-specific challenges. Adaptations to overcome these limitations included the use of same-species fetal fibroblasts as feeder cells, trialing feeder-free culture systems, serum-free media, altered growth factor cocktails, using a culture environment of 10% O_2_ and reduction of temperature from 37°C to 35°C [169]. These conditions maintained bovine germ cell colonies, 22% of which were actively dividing, however, a gradual decline in cell numbers led to loss of cultures by 8 weeks [169]. In a similar study, Zhang et al. [170] found that neonatal autologous Sertoli cells supported porcine undifferentiated spermatogonia better than mouse SIM embryonic fibroblast-derived feeder cells or adult Sertoli cells, and conditions, including serum-free media, growth factor supplementation (GDNF, GFRA1, FGF2, and IGF1) and incubation at 35°C maintained the cultures for up to 2 months before UCHL1 expression rapidly declined.

To our knowledge, very limited research has been conducted on *in vitro* cultivation of SSCs from wildlife species, however, one study has reported the successful propagation of tree shrew SSCs [171]. The authors described a culture system for the long-term expansion of THY1-MACS isolated undifferentiated spermatogonia, using adult tree shrew Sertoli cells as feeder cells. RNAseq analyses were used to identify growth factor-driven self-renewal pathways that might be unique to tree shrew SSCs, thereby identifying the Wnt/β-catenin pathway. Subsequently, supplementation with recombinant WNT3A enhanced the survival of tree shrew SSCs, at least during the initial passages of primary culture. This was validated through SSC transplantation into adult tree shrews sterilized via cytotoxic treatment, demonstrating restored spermatogenesis and the production of transgenic offspring [171]. The successful maintenance and characterization of undifferentiated SSCs from a species other than rodents or domestic animals provides significant promise for future applications in wildlife, spanning from species conservation to gene editing and ARTs.

## Spermatogonial transplantation

The spermatogonial transplantation technique was first reported in 1994, where microinjection of donor germ cells into the seminiferous tubules of infertile recipient male mice was used to identify SSCs by way of their capacity to self-renew and drive spermatogenesis [172, 173]. This initial approach used testis cell suspensions obtained from transgenic mice expressing the *LacZ* reporter gene encoding β-galactosidase. In tracking the colonization of LacZ expressing donor cells, this study provided proof-of-principle that SSCs injected into the lumen of the seminiferous tubules can migrate to the basement membrane, crossing the blood-testis barrier formed by Sertoli cells, and engraft in available niches. It has subsequently been demonstrated that the number of transplanted donor SSCs is directly proportional to the number of donor-derived colonies of spermatogenesis at three months post-transplantation, however colonization efficiency has been calculated to be only 10–15% [174–176]. Regardless of poor colonization efficiency, following spermatogonial transplantation in mouse models, donor-derived spermatozoa appear by 2 months post-transplantation [172]. These spermatozoa are morphologically and functionally normal, capable of fertilizing an egg and producing fertile progeny carrying the donor haplotype.

The development of the spermatogonial transplantation technique was revolutionary for the field of SSC research and has been adopted as the “gold-standard” for quantitatively assessing SSC content within a heterogeneous population of cells. However, in concert with its utility in discovery-based science, this technique also demonstrates significant potential for clinical applications in addressing certain types of male infertility, particularly when combined with gene therapy in the case of congenital or hereditary disorders [177–179]. In conservation efforts, this technique could play a vital role in pipelines designed to reintroduce genetic diversity into endangered wildlife populations. This could be achieved through the transplantation of biobanked SSCs from a deceased individual, or an individual from a geographically distinct population, into a recipient male who would then be released to disseminate these genetics through natural mating.

### Generation of sterile transplantation recipients

Success of the spermatogonial transplantation technique relies on several key factors, one of which is microinjection into a recipient testis that has “empty” niches for the donor SSCs to colonize. In mouse models, recipients are commonly generated via treatment with sub-lethal doses of the alkylating chemotherapeutic agent, busulfan, which eradicates endogenous germ cells [180]. Alternatively, genetically sterile mutant mouse strains have also been used, such as the White-spotting mouse strain [172]. In livestock species, the depletion of endogenous germ cells has most commonly been achieved through testis irradiation [181, 182]; however, busulfan-based methodologies have also been employed [183]. Importantly, data from rodent studies has shown that complete elimination of endogenous germ cells is not a prerequisite for establishing donor spermatogenesis [50, 184–186]. Indeed, proliferation and differentiation of donor spermatogonia could be enhanced by residual endogenous spermatogenesis, aiding the maintenance of a healthy testicular microenvironment in the recipient animal [184].

A limitation for spermatogonial transplantation in wildlife species is the production of same-species recipients. Although this has been successfully achieved in the tree shrew by pre-treating transplantation recipients with busulfan [171], the risk of systemic toxicity limits the utility of this approach in vulnerable and endangered species [187]. Similarly, generating genetically sterile recipients for endangered species will not be feasible. In such cases, irradiation of recipient testes would likely be the most viable option.

### Allogeneic versus interspecies transplantation

In addition to allogeneic SSC transplantation, interspecies spermatogonial transplantations (i.e., xenotransplantation) have also been reported. The first such experiment introduced rat SSCs into the testes of busulfan-treated immunocompromised mouse [188]. Here, donor cells were able to colonize recipient tubules and establish spermatogenesis, producing morphologically normal and fertilization competent spermatozoa that produced healthy progeny [189]. Equivalent results were also demonstrated in the reverse, with mouse SSCs successfully reconstituting spermatogenesis after transplantation into rat testes [190, 191]. Other combinations of interspecies SSC transplantation have also been performed, namely hamster, rabbit, cat, dog, pig, cattle, horse, marmoset, baboon, macaque, and human SSCs into immunocompromised mouse recipients; suggesting that xenogeneic spermatogenesis can be reconstructed with phylogenetically closely related donor and recipient species (reviewed in [192, 193]). Importantly, in all non-rodent species, xenotransplantation failed to induce complete spermatogenesis; however, donor SSCs (rabbit, cat, dog, pig, cattle, horse, marmoset, baboon, macaque, and human) were still able to colonize and proliferate in mouse seminiferous tubules for long periods, regenerating the undifferentiated pool [194–198]. This demonstrates that stem cell recognition and colonization are highly conserved among species, which highlights the plasticity of the SSC niche in accepting various germline cells.

Xenotransplantation could have significant utility in wildlife conservation, whereby transplanting SSCs from endangered species to closely related domestic hosts could enable the production of fertilization-competent donor spermatozoa for use in ARTs. The success of this approach has been demonstrated in feline species, where SSCs from wild felids (ocelots) were transplanted into domestic cat recipients sterilized through busulfan treatment or local irradiation [199]. This resulted in the successful colonization and differentiation of donor cells, with ocelot spermatozoa being observed in the cat epididymis 13 weeks following transplantation. Although the viability and fertilization capacity of ocelot sperm was not determined, this study demonstrates the feasibility of using closely related, non-threatened species as recipients for the propagation of male germ cells from endangered species.

Phylogenetic distance between the SSC donor and recipient is likely to be the limiting factor in this approach. While the estimated divergence time between the ocelot and domestic cat is ~8 million years (MY) [200], no definitive cutoff has been established. If we were to extend this method to the conservation of endangered Australian marsupials, such as the koala, mountain pygmy possum (*Burramys parvus*), northern hairy-nosed wombat (*Lasiorhinus krefftii*), brush-tailed bettong (*Bettongia penicillata*), etc., we would require a non-endangered related recipient whose endogenous SSCs have been eliminated through appropriate pretreatment. Recipient species that could be considered include some common macropod species like the eastern grey kangaroo (*Macropus giganteus*) and tammar wallaby (*Notamacropus eugenii*) both of which have stable populations in the wild and an IUCN conservation status of “least concerned”. Furthermore, the estimated phylogenetic divergence between the macropods and each of the exemplified endangered species is 53, 45, 53, and 21MY, respectively [201–204]. Considering the koala, potential closely related recipients include the common wombat (*Vombatus ursinus*) and southern hairy-nosed wombat (*L. latifrons*). These species show a genetic divergence of 23.38% and a phylogenetic distance of ~35MY from the koala [202, 205, 206]. The southern hairy-nosed wombat could also serve as a potential model species for its critically endangered northern counterpart, with an estimated divergence time of 12.6MY [204, 207]. On the other hand, recent advancements in conservation biology have led to the development of a laboratory model using the fat-tailed dunnart, a small marsupial roughly the size of a mouse [208]. Experimental colonies of this species have been successfully established aiming to enhance our understanding and protect the unique biology of Australian marsupials. Despite the substantial estimated phylogenetic divergence of ~61MY between the fat-tailed dunnart and the koala, mountain pygmy possum, northern hairy-nosed wombat, and brush-tailed bettong [204, 206], the existing colonies of this small marsupial offer a potentially valuable resource within this context. Importantly, although xenotransplantation holds great promise as a conservation tool, successful adaptation of this technique across species would necessitate extensive *in silico* and *in vivo* experimentation.

Finally, it is worth mentioning that, due to the current lack of functional assays available to identify and quantify non-rodent SSCs, xenogeneic transplantation assays into immunocompromised mice have been implemented for assessment of stem cell activity from a number of species [170, 194–196, 209]. Interestingly, these studies have shown that the exogenous factors promoting non-rodent SSC survival and proliferation present in the mouse SSC niche are conserved among mammals, whereas the factors involved in differentiation appear to be species-specific. It has been argued that transplantation of Sertoli cells along with SSCs from the same species could reestablish the necessary microenvironment required to support differentiation of the donor stem cells and in that way generate a system for the functional assessment of non-rodent SSCs [192]. This is an important consideration for the future development of these techniques in wildlife species.

### Microinjection technique and identification of donor cells in the recipient

Due to the species-specific anatomy of the testis, modifications to the transplantation procedure developed in mice have been necessary for use in non-rodent animals. In rodents, the most widely adopted technique is single microinjection through the efferent duct, which is accessed via transabdominal surgery [190, 191]. However, alternate injection sites include the rete testis [210], and directly into the seminiferous tubule [173]. Since the testicular architecture of larger animals does not permit injection into the efferent ducts or directly into the seminiferous tubules, microinjection of donor SSCs into the rete testis has been shown to be the most favorable approach [211]. This method was further optimized by the application of ultrasonography to localize the rete testes and guide the injection needle, thereby eliminating the need for open surgery. This technique has since been successfully performed in various species including primates, pigs, goats, sheep, and cattle [181, 212–214]; however, it still presents a challenge in equids [215] and will likely present a challenge for small marsupial species.

Additionally, in contrast to rodents, transgenic strains expressing reporter genes are not available in livestock and wildlife species. Therefore, different approaches have been used to identify donor-derived colonies of spermatogenesis following transplantation, which have primarily involved genetic labelling of donor SSCs. Methodologies that have been trialed include viral transduction of donor SSCs followed by genotyping of the ejaculate to detect transgenic sperm [216, 217], as well as screening for donor-specific microsatellite DNA in semen samples [107, 218, 219]. A key limitation to these approaches is the inability to determine whether the source of detected donor DNA is of somatic or germ cell origin. Thus, to prove the feasibility of SSC transplantation in non-rodents, generation of progeny with donor SSC haplotype through *in vitro* fertilization (IVF) or ICSI is required. Nonetheless, these studies provide valuable evidence for the successful application of SSC transplantation in non-rodent species.

## Testis tissue grafting

Testis tissue grafting involves ectopic (grafting to a location other than the testis) or orthotopic (scrotal grafting) transplantation of tissue between or within individuals. This includes autografts (same individual), allograft (same species), and xenografts (different species), where the recipient functions as a living bio-incubator [220]. This creates a controlled environment with restored blood and endocrine supply for the development of the grafted tissue.

While the development of spermatogonial transplantation techniques represented a major breakthrough in SSC research, this technique has practical and financial limitations, including incomplete spermatogenesis in interspecies transplantations as a consequence of incompatibility between the donor cells and the host testicular microenvironment [194–197]. Testis tissue grafting overcomes this issue by transplanting donor spermatogonia along with their surrounding somatic compartment [221]. Further, unlike with spermatogonial transplantation, the fertility status of the host animal does not impact the spermatogenic success of the grafted tissue [222]. Additionally, the simple injection of testis tissue using tumor transfer needles renders the grafting procedure significantly less invasive than spermatogonial transplantation [223], and is considerably less complex. Together, these factors highlight that testis tissue grafting may be a useful tool when dealing with non-model species [224]. Albeit one limitation is the tendency for graft degeneration with the use of adult tissue [225, 226], as opposed to immature tissue from neonatal and prepubertal donors, which demonstrates better survival and spermatogenic capability [227, 228]. Furthermore, unlike spermatogonial transplantation where *in vivo* spermatogenesis facilitates natural mating, generating offspring with graft-derived sperm requires the use of ARTs.

### Testis tissue grafting in human medicine

Successful xenografting studies have primarily used immunodeficient mice as recipients (reviewed in [229]). However, it is notable that xenografted adult human testicular tissue has very limited survival of spermatogonia in nude mice [225, 230]. Xenotransplantation of prepubertal human tissue achieves improved seminiferous tubule integrity; however, spermatogonial survival has again been reported to be very limited at 4- and 9-months post-transplantation [231]. Similar results were observed following ectopic grafting of immature marmoset testicular tissue into nude mice, along with poor androgen production in both human and marmoset xenografts [230, 232]. When immature human testicular tissue was orthotopically transplanted into the scrotum of immunodeficient mice after castration, a short-term evaluation (3 weeks post-grafting) revealed well preserved tubular structures but significant germ cell loss [233]. Subsequent long-term (6 months post-grafting) assessment confirmed the survival and proliferation of spermatogonia in grafts from cryopreserved immature human tissue; however, spermatogenic development beyond the pachytene stage was not observed [234]. Complete *ex vivo* spermatogenesis in the marmoset was finally reported when immature tissue was grafted under the testicular parenchyma of immunodeficient mice [235], likely facilitated by favorable temperature regulation in the scrotum along with paracrine support, however, these results were not recapitulated in human intratesticular xenografts, highlighting the need for further research [236, 237]. While non-human primates with closer phylogenetic distance to humans might offer a more suitable pre-clinical model than rodents, practical and ethical considerations, including regulatory concerns, could pose challenges to their use [238].

Despite advancements in xenografting, its application in clinical settings raises concerns due to the risk of zoonoses, and ethical considerations associated with generating human germ cells in animals [239]. Thus, autografting is the leading contender for use in the clinical space. The primary drawback of autografting, much like autologous SSC transplantation, lies in the potential reintroduction of malignant cells from the testis tissue fragments. To tackle this issue, a strategy similar to ovarian tissue transplantation could be adopted to the testes, involving the estimation of the risk of metastases to the reproductive organs based on the primary disease type and screening for disease-specific molecular markers [240].

### Testis tissue grafting in wildlife conservation

Much like SSC transplantation, grafting of bio-banked testicular tissue could have significant utility in maintaining biodiversity for wildlife species by facilitating the recovery of mature sperm from valuable individuals and reintroducing their unique genetics into the population at a later date (Figure 4). However, as discussed, the use of testis grafting may be restricted to immature donors. This presents a limitation for rare and endangered species, where availability of immature tissue is extremely sporadic. On the other hand, in situations where immature male animals require euthanasia due to disease or road accidents, this technique becomes an invaluable tool for conserving genetic diversity. For example, recent studies showed that autologously grafted [108] and xenografted [241] fresh and cryopreserved prepubertal testicular tissue from Rhesus macaques underwent complete spermatogenesis, leading to recovery of mature, fertilization-competent sperm, and the subsequent birth of healthy babies through ICSI.

In endangered wildlife species, an initial attempt to preserve fertility through xenotransplantation was reported in the banteng (*Bos javanicus*), with immature testicular tissue being grafted under the back skin of castrated immunodeficient male mice [242]. Histological examinations at 3, 6, 9, 12, and 15 months after grafting revealed that banteng spermatogenesis was initiated in the xenografts; however, only advanced to the stage of pachytene spermatocytes, potentially due endocrine incompatibilities. Beyond this, however, testicular tissue from the common ferret (*Mustela furo*) has been successfully xenografted onto castrated nude mice, producing differentiating germ cells, mature sperm, and physiologically relevant levels of testosterone [243]. These studies present potential pipelines for the use of xenotransplantation to produce mature spermatozoa in other wildlife species.

## *In vitro* spermatogenesis

Although spermatogonial transplantation and testicular tissue grafting hold promise as methods for restoring spermatogenesis and recovering mature spermatozoa in many species, ethical, logistical, and financial restraints exist with the use of animal models. This could be circumvented with the use of *in vitro* systems for SSC differentiation and downstream spermatogenesis, thereby negating the need for animal hosts. However, as with *in vitro* maintenance of undifferentiated spermatogonia, many extrinsic regulatory signals are involved, albeit with the added layer of complexity of orchestrating not only spermatogonial differentiation, but also meiosis, and the drastic morphological transformation that occurs during spermiogenesis. These regulatory factors include cytokines, growth factors, nutrients, and hormones that could either act directly on the developing germ cells or indirectly through action on the supporting somatic cells in the testis. Not surprisingly, the identification of crucial factors and conditions to support spermatogenesis *in vitro* remains elusive in most species.

### Organotypic culture system

A commonly employed method for *in vitro* spermatogenesis (IVS) is organ culture involving the *in vitro* cultivation of whole fragments of testicular tissue, with the rationale that spermatogenesis can resume *in vitro* with the support of the natural microenvironment. In 2011, Sato et al. made a substantial breakthrough in directing mouse IVS by refining a gas–liquid interface method that had previously supported the production of pachytene spermatocytes [244, 245] by reducing incubation temperature to 34°C and using alphaMEM medium with knockout serum replacement (KSR) instead of FBS [246, 247]. This modification allowed the generation of round and elongated spermatids, which resulted in the production of fertile offspring through ROSI and ICSI [248]. Furthermore, a follow-up study demonstrated that the nuclear quality of mouse spermatozoa created using this method closely resembled that of *in vivo*-generated controls [249].

More recently, the same research group achieved IVS for the first time in the rat [250]. Here, the authors aimed to unravel the metabolic dynamics of the cultured testicular tissue by monitoring changes in the concentrations of major culture medium components over 28 days. They observed dramatic changes in concentration of 50 substances, with seven of them (glucose, pyruvate, aspartic acid, glutamic acid, valine, leucine, and isoleucine) showing a significant decrease over time. Supplementing the culture medium with these components, along with lowering oxygen concentration in culture to 15% [251], resulted in consistent production of elongated spermatids, which subsequently gave rise to healthy and fertile offspring through ICSI [250]. This study underscores the critical role of cellular and tissue metabolism in spermatogenic success and provides the foundation for the development of IVS systems in other species through application of metabolic analysis and corresponding (species-specific) modifications to the culture medium.

Finally, the organ culture system has also achieved complete IVS in non-rodent species such as goats, resulting in the generation of elongated spermatids from either fresh or vitrified-warmed testicular tissue [135, 252]. However, no functional assessment of the produced haploid cells has been performed leaving their fertilization competence and potential to produce viable offspring undetermined.

### 3D and 2D culture systems

Transitioning from traditional organ culture to three-dimensional (3D) culture systems for testicular somatic and germ cells represents a significant advancement towards achieving more defined conditions for IVS. 3D cultures enable crucial cell–cell and cell-extracellular matrix interactions, mimicking the testicular architecture. Initially, research in this area showed promise in generating tubule-like structures from neonatal mouse testis cell aggregates cultured on an agarose gel at a gas–liquid interface. These tubules contained cells differentiated to the stage of pachytene spermatocytes and a small number of haploid cells [253]. The first *in vitro* generation of functional haploid spermatids from human SSCs was reported using a 3D-induced system [254]. This system involved co-culturing GPR-125-MACS isolated spermatogonia and mitotically inactivated Sertoli cells in 3D Matrigel with defined medium (DMEM/F12 supplemented with 10% KSR, retinoic acid, SCF, testosterone) at 34°C and 5%CO_2_. The resultant round spermatids were assessed according to a “gold standard” criteria for *in vitro*-derived gametes [255], including chromosome content, synapsis and recombination, epigenetic imprinting, fertilization, and development capacity. More recently, a 3D culture system consisting of a hybrid hydrogel of agarose and laminin was employed for *in vitro* differentiation of human SSCs, following which the formation of mature spermatozoa was reported [256]. Progress in this area has been partially attributed to the role of laminin as a key basement membrane component and its ability to bind growth factors in the culture medium and increase their local concentration [257]. Furthermore, its affinity for binding SSCs (via ITGA6/B1) [258, 259] and Sertoli cells [260] accelerated cellular assembly and reduced apoptosis, particularly important during the initial 24 h of the culture period [261]. While several alternative 3D culture systems have been proposed, utilizing materials such as collagen matrix, soft agar, hanging transwell inserts or microwells (reviewed in [262]) they have yet to report convincing evidence of meiosis progression or functionality of haploid cells.

Achieving successful IVS carries crucial implications for both male infertility treatments and wildlife conservation. In the context of human medicine, generation of mature sperm *in vitro* holds promise for men suffering from azoospermia or different genetic causes of spermatogenic failure. It also offers an alternative to SSC transplantation for preserving fertility in prepubertal male cancer patients, mitigating the risk of malignant reintroduction. However, comprehensive analysis of human haploid cells generated *in vitro* along with a thorough examination of offspring produced from *in vitro*-differentiated haploid cells of model animals is imperative before implementing IVS in a clinical setting. Additionally, spermatogonial extraction combined with IVS, followed by ARTs, present a significant opportunity for conserving the genetic diversity of endangered wildlife species, thereby preventing their extinction. These methods prove invaluable when natural reproduction is hindered by injuries or the stresses of captivity. Importantly, the lack of fundamental knowledge on the early stages of wildlife spermatogenesis poses a challenge in evaluating the quality of in vitro-derived gametes from these species, particularly the efficacy of meiosis. Therefore, the assessment of “gold standard” parameters in the generated gametes (e.g., chromosomal content and organization, rates of genetic recombination, ability to generate euploid offspring) [255], necessitates the initial characterization of their diploid precursors.

## Conclusions

While it is true that the goals of spermatogonial stem cell technologies differ between the realms of human medicine and wildlife conservation, the tools for achieving these goals are largely aligned. In human medicine, SSC technologies are primarily being developed to reverse infertility, particularly in survivors of childhood cancers. Contrastingly, SSC biobanking in wildlife conservation aims to retain genetic diversity to ensure species adaptiveness, integrity, resistance to disease, and reproductive fitness: holding particular utility for species such as the koala, whose mature spermatozoa do not survive current cryopreservation strategies. In either setting, however, we propose that these outcomes may be achieved through cryostorage of testis tissue, followed by SSC transplantation, tissue grafting, or *in vitro* spermatogenesis.

In this review, we have highlighted the gaps-in-knowledge surrounding the basic biology of spermatogonia from non-model species, including niche factor requirements, metabolic preferences, surface markers, and proliferative dynamics. However, upon a preliminary assessment of gene expression of SSCs from model species (mice), humans, and marsupials (opossum), we have demonstrated that, reassuringly, the basic building blocks of SSC biology (growth factors and their downstream signal transduction pathways, and core transcription factors) appear to be conserved between species. Thus, there is cause for cautious optimism that SSC-based techniques in development for human medicine will be amenable to translation for the conservation of less well studied species, such as the koala. Regardless, there is a clear need for additional research to bolster our understanding of cross-species SSC biology.

Herein, we have also reviewed the benefits and limitations associated with different SSC technologies, particularly for wildlife conservation. For example, we have identified that testicular tissue grafting may be the preferred approach when pre-pubertal tissue is available, while spermatogonial transplantation, although more complex, holds potential for utilizing SSCs from pre-pubertal or adult testes to disseminate donor genetics via natural breeding. *In vitro* spermatogenesis has the furthest to go in terms of scientific development for non-model species and relies solely on ARTs for dissemination of donor genetics, however, would undoubtedly hold utility in that the requirement for a “recipient” individual is negated. Notably, while testis tissue grafting and IVS hold significant promise, they will not be feasible without complementary research into oogenesis / oocyte retrieval, as well as refinement of ART techniques (e.g., ICSI), in vitro culture of embryos, and embryo transfer. Thus, species-specific research into these processes is equally crucial to bolster the feasibility of SSC technologies for endangered species conservation.

In conclusion, although reproductive technologies utilizing SSCs possess a degree of complexity, they hold particular promise for populations in which cryostorage of mature gametes is not possible. Through the lens of wildlife conservation, we propose that SSC pipelines should be developed in parallel with other reproductive technologies to maximize our capability to prevent species extinction. Of course, such strategies will be in vain if a concerted effort is not made to protect the environments and habitats in which these species reside [35, 41–49, 53–56, 80, 263–274].

## Supplementary Material

Supplemental_Dataset_legends_ioae109

## Data Availability

Data utilized in this review have been previously published and can be accessed from the GEO database under the following accession numbers: GSE109033 (adult mouse spermatogenic cells) [36], GSE109037 (adult human spermatogenic cells) [36], and E-MTAB-11072 (grey short-tailed opossum germ cells) [30].
