# Supplementary material for: Spermatogonial stem cell technologies: applications from human medicine to wildlife conservation
Source: Biol Reprod. 2024 Jul 12;111(4):757–79. doi: 10.1093/biolre/ioae109 (PMC11473898; doi:10.1093/biolre/ioae109)
Supplement: Supplemental_Dataset_legends_ioae109 [file supplemental_dataset_legends_ioae109.docx]

**Supplemental Dataset legends**

**Supplemental dataset S1:** Spermatogonia identifiers, conserved genes, and gene ontology analyses related to data presented in Fig. 2.
